# Are parenting practices associated with the same child outcomes in sub-Saharan African countries as in high-income countries? A review and synthesis

**DOI:** 10.1136/bmjgh-2018-000912

**Published:** 2018-12-27

**Authors:** Alison M Devlin, Daniel Wight, Candida Fenton

**Affiliations:** 1 MRC/CSO Social and Public Health Sciences Unit, Institute of Health and Wellbeing, University of Glasgow, Glasgow, UK; 2 Cochrane Vascular, Usher Institute of Population Health Sciences and Informatics, University of Edinburgh, Medical School, Teviot Place, Edinburgh, United Kingdom

**Keywords:** parenting, sub-Saharan Africa, child and adolescent health, transferability, complex interventions, low income countries

## Abstract

**Introduction:**

There is increasing interest in the transferability of parenting interventions from high-income countries (HICs) to low-income countries (LICs) in order to improve child development and health outcomes. This is based on the premise that associations between parenting practices and child outcomes are similar in both settings. Many parenting interventions in HICs are evidence-based, but less evidence exists on associations of parenting practices with child outcomes in LICs, in particular, sub-Saharan African (SSA) countries. This review synthesises evidence on the association of parenting practices with child outcomes in SSA in order to compare findings with those from HICs.

**Methods:**

We searched electronic databases—Web of Science, ASSIA, Embase, IBSS and PsycINFO—to identify studies from SSA that reported quantitative associations between parenting practices and child health or psychosocial outcomes (eg, sexual and reproductive health (SRH), mental health, conduct disorders). Due to inconsistent conceptual framing of parenting across studies, we used a modified version of the international WHO classification of parenting dimensions to guide synthesis of the results.

**Results:**

Forty-four studies met our inclusion criteria. They were conducted in 13 SSA countries and included cross-sectional and longitudinal studies, and were predominantly descriptive studies rather than intervention research. Synthesis of results showed that associations between patterns of parenting (‘positive’/‘harsh’) and child outcomes (including SRH, mental health and conduct disorders) in studies from SSA were broadly similar to those found in HICs.

**Conclusions:**

These findings suggest that the impacts of parenting practices on child outcomes are similar across contrasting global regions and, therefore, parenting interventions from HICs might be successfully transferred to SSA, subject to appropriate adaptation. However, this review also highlights the paucity of evidence in this area and the urgent need for higher quality studies to confirm these findings to help develop effective parenting interventions in SSA.

## Introduction

There is great interest in parenting interventions and their potential transfer from high-income countries (HICs) to low-income and middle-income countries (LMICs) in order to improve child development and health outcomes.^1–3^ These outcomes include sexual and reproductive health (SRH); mental health; maladaptive behaviours and conduct disorders.^4^ Furthermore, the WHO (2010) launched the ‘Parenting for Lifelong Health’ initiative to develop interventions in LMICs, including sub-Saharan Africa (SSA), thereby endorsing the key role of parenting in healthy child development.^5^ Gardner *et al* previously found evidence that parenting interventions can be successfully transferred across culturally distant regions, but this study only included one LMIC and no SSA countries.^3^ Most parenting interventions (eg, ‘Incredible Years’, ‘Triple-P’) have a theoretical evidence base that was built in HICs.^6–9^ Consequently, the transfer of interventions from HICs to LMICs is based on the premise that *associations between* parenting practices and child outcomes are similar across regions. But is what is good for children in HICs also good for children in LICs? Although other sociocultural features are also important for successful intervention implementation, studies investigating associations between parenting practices and child outcomes are only now emerging from LICs, including SSA, and the evidence base is limited.^10–12^

The conceptual framing of parenting, as theorised in the Western literature, typically adopts a (i) parenting ‘styles’ or (ii) parenting ‘dimensions’ approach.^13^ The seminal work by Baumrind^14–17^ on the dynamic nature of parent–child interactions led to a ‘parenting style’ typology based on the constructs of ‘warmth’ and ‘control’ and documented three main styles: authoritarian (low warmth, high control); authoritative (high warmth, high control) and permissive (high warmth, low control). Others have disaggregated ‘parenting styles’ and focused on aspects of parenting practice or dimensions including parent–child connection, behaviour control, communication and parental monitoring.^18 19^ However, there is some overlap in the theoretical approaches adopted.^20^

The research evidence base in HICs has documented associations between parenting styles and a range of child and adolescent outcomes.^21^ Authoritative parenting has been associated with positive outcomes including protection from poor mental health and substance use, as well as improved educational outcomes.^22 23^ The authoritarian parenting style is associated with negative outcomes including poor self-esteem and depression.^24^ Similarly, a strong evidence base exists on parenting practices or dimensions. There is evidence for the association of parent-child connection (warmth) and protection from risky sexual behaviours,^25^ poor mental health,^26 27^ conduct problems, antisocial behaviour (ASB) and substance use.^25–27^ Conversely, *a lack of* parental connection is associated with poor adolescent mental health and poor child adaptive functioning.^28–30^ Studies in HICs have also shown the important role of parental monitoring on protection from poor outcomes including risky sexual health behaviours.^18 31 32^ In addition to ‘positive’ parenting that contributes to healthy child development, there is a body of evidence on parenting practices that are harmful.^33^ Harsh parenting practices also include dimensions of child maltreatment. First, parental psychological control (eg, cold and hostile behaviour), which has been associated with poor adolescent mental health and conduct disorders.^34^ Second, severe physical punishment as discipline, which has been associated with child internalising and externalising behaviours.^35 36^

There are cultural differences in child development in SSA countries, which also differ markedly from HICs in terms of socioeconomic context.^37^ There is also evidence to suggest that there are cultural differences in the way children respond to parenting practices.^35 38 39^ However, a number of these studies were based on comparison across ethnic groups in one country.^39 40^ For example, Lansford *et al* reported harsh parenting was associated with more externalising behaviours in European American compared with African-American adolescents.^40^ There is ongoing debate surrounding physical discipline and corporal punishment of children,^35 36 41^ with some authors suggesting it is less harmful in countries where it is culturally normative,^42^ such as several SSA countries, where the prevalence rate remains high.^43^ Although some cross-national studies have been conducted to compare cultural variations in child outcomes in response to parenting, few have included SSA countries.^42^

The present review aims to answer the following research question: ‘*Are the associations between parenting practices and child outcomes in SSA similar to those that have been well documented in HICs?’* In so doing, we hope to contribute to the quantitative evidence on how ‘positive’ and ‘harsh’ parenting practices relate to child/adolescent outcomes in SSA countries in order to help clarify the suitability of transferring interventions from HICs to SSA.

## Methods

### Literature search

The search strategy was designed to capture studies with data on the association of any parenting practices (or styles) with a range of child health outcomes in SSA countries and was conducted by an experienced Information Scientist (CF). Five bibliographic databases were searched: Web of Science, IBSS, ASSIA, Embase and PsycINFO. These databases cover a range of subject areas including psychological, social and clinical sciences and a range of journals relevant to the review. Searches were conducted across all fields, including title, abstract and index terms. Search statements were adapted for each database search (table 1) according to the number of relevant references they retrieved. Initial hits (n=3974) were screened (by CF) to exclude those clearly irrelevant and duplicates. Further potentially relevant references were retrieved by identifying papers cited in key papers.^44^ All potentially relevant papers (n=150) were exported to Endnote Bibliographic Management software (V.7.4) for detailed screening. All searches were run during February and May 2016 by CF.

**Table 1:** Search statements *included*

“Adverse childhood experiences” AND Africa* “Authoritarian Parenting” AND Africa “Authoritative Parenting” AND Africa “child abuse” AND Africa* “child abuse” AND parent* AND Africa “child parent relation” AND Africa* “child parent relation*” AND Africa “Childhood maltreatment” AND Africa* “Childrearing Practices” AND Africa “Corporal punishment” AND Africa* “emotional abuse” AND Africa* “emotional neglect” AND Africa* “family structure” AND “Saharan Africa” “harsh punishment” AND Africa* “Parent Child Communication” AND Africa “Parent Child Relations” AND Africa “Parental Bonding Instrument” AND Africa*	“Parental Involvement Scale” AND Africa “Parental Involvement” AND Africa “parent-child communication” AND “sub-Saharan Africa” “Parenting Style” AND Africa “parenting style*” AND Africa* “Permissive Parenting” AND Africa “physical punishment” AND Africa* attachment AND Africa AND parent* Coparenting AND Africa Parent* AND Africa Parenting AND Africa parenting AND Africa AND aspiration parenting AND Africa AND college parenting AND Africa AND education* parenting AND Africa AND goal parenting AND Africa AND hope

### Article screening and selection

**Figure 1:**
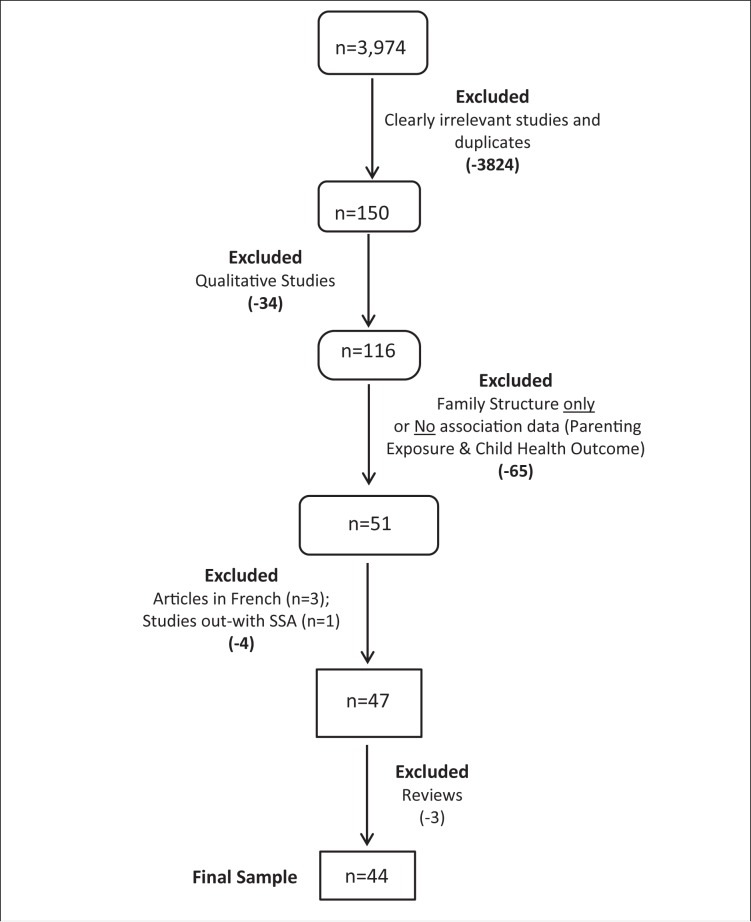
Flow diagram of article screening pathway.

All potentially relevant bibliographic records (n=150) were retrieved to determine if they included quantitative data on the association of a parenting exposure with a child outcome in SSA. The initial sample included randomised controlled trials (RCTs), large-scale national studies, qualitative studies, social narratives and case studies. The stages involved in the screening process are summarised in a flow diagram (figure 1). The inclusion and exclusion criteria are summarised in box 1.

Box 1. **Inclusion and exclusion criteria**

**Inclusion criteria**

- Published (years 1990–2016)
- Peer-reviewed articles
- English-language
- Conducted in sub-Saharan Africa
- Association data between aspect of parenting (exposure) and child/adolescent health (outcome)
- Child outcomes that have been studied in high-income countries

**Exclusion criteria**

- Association data on aspects of family structure *only* and child health outcomes
- Inappropriate outcomes: eg, highly technical issue of child self-identity or financial ‘health’

AD was the main reviewer, and regular review meetings were held between AD and DW to ensure consistency of inclusion and exclusion criteria. A subsample (approximately 10%) was reviewed by the second reviewer (DW) in a blinded manner and decisions were largely in agreement (12/15) which served as a further indicator of consistency. It was decided that if decisions could not be finalised by AD and DW then a third researcher would arbitrate but this was unnecessary. Screening sequentially, we first excluded *qualitative-only* studies (n=34: 32 articles and 2 book chapters). Next, we excluded all studies that had only investigated family structure (and not parenting practices), as well as those that did not include any associations between parenting exposure(s) and outcomes (n=65), resulting in 51 studies remaining (figure 1). We then excluded articles in French (n=3) and one study that was conducted outwith SSA. Finally, we excluded three reviews since our search had identified the relevant articles within them. This resulted in a final sample of 44 studies that met our inclusion criteria.

### Data extraction and analysis

**Figure 2:**
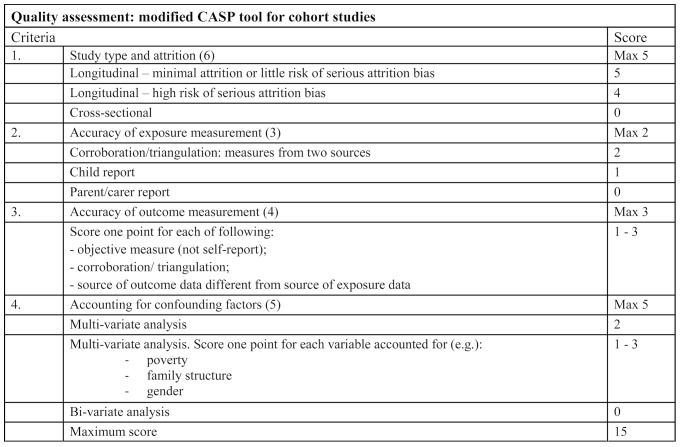
Modified CASP (for cohort studies) tool.^45^ SSA, sub-Saharan Africa.

Data extraction was conducted by the main reviewer (AD) using a template specifying study authors, location, design, sample size, sampling procedure, parenting exposure, child outcomes and main findings. Studies were quality assessed using *a modified version* of the CASP tool for Cohort studies.^45^ Assessment criteria were (1) study type, (2) attrition, (3) accuracy of exposure measurement, (4) accuracy of outcome measurement and (5) whether accounted for confounding factors (figure 2). Studies were scored for each criterion on a four-point scale, these were summed and the sample dichotomised between ‘poor-medium’ quality (score ≤4) and ‘higher’ quality (score >4). We did not exclude studies on the basis of quality but appraised them in order to take account of study quality in the analysis.

The sample of studies was heterogeneous and complex, often with poor conceptual framing of ‘parenting’ styles or dimensions across studies and the use of broad parenting constructs. Furthermore, there was marked inconsistency in the tools used to measure parenting across studies. Therefore, results were initially structured by outcomes since these constructs corresponded to the original studies. There was clustering according to six outcomes; parenting and child (or adolescent): (1) SRH, (2) adaptive functioning/ development, (3) educational outcomes/academic achievement, (4) mental health/depression, (5) ASB/conduct disorders/delinquency and (6) substance use (alcohol and tobacco). The data are summarised in online supplementary table 1.1-1.6 in, and we provide a narrative analysis of the findings in the ‘Results’ section.

10.1136/bmjgh-2018-000912.supp1 Supplementary data

### Data synthesis

Since constructs of exposure (ie, parenting practices) were so varied, we mapped each study using a modified version of the WHO categories of parenting roles (WHO, 2007; table 2).^46^ Since the WHO (2007) parenting roles resulted from a review of international literature and consensus exercise with international experts, we considered this to be a suitable generic, culturally appropriate typology to guide data synthesis. Each study was coded using this modified typology in order to synthesise the results according to parenting role (see column 4 in online supplementary table 1.1-1.6 and table 3). In a few studies, parenting styles were clearly measured (*and coded as such*), and in a small number of studies, parenting styles *and* individual parenting roles were both measured. To establish if results differed according to the quality of studies, we split the sample between ‘poor-medium’ (score ≤4) quality studies and ‘higher’ (score >4) quality studies. This is presented in two additional summary tables (table 4 and table 5). The main findings, as summarised in the results (online supplementary table 1.1-1.6) and synthesised in tables 3–5, were statistically significant (at p<0.05) unless stated otherwise.

**Table 2:** Modified WHO parenting dimensions (adapted from WHO^46^)

WHO role/dimension	Adapted role/dimension	Description
Connection	Connection	Parental warmth, affection and care. Conveys that child is loved and accepted.
	Connection: general communication	Positive communication expressing support and encouragement.
	Connection: SRH communication	Communication specifically about SRH issues.
Behaviour control	Positive behaviour control	Positive regulation, limit setting and monitoring.
	Negative behaviour control	Harsh punishment, especially corporal punishment, physical or psychological punishment.
Modelling appropriate behaviour	Role modelling	Role modelling of risky or protective health related behaviours or attitudes.
Provision and protection	Provision and protection	Provision of own resources, accessing external resources (networks) for example, for schooling/ education.

SRH, sexual and reproductive health.

**Table 3:** Synthesis of results according to parenting exposure: total sample (n=44)

Parenting role	Sexual and reproductive health	Mental health	Educational achievement	Adaptive functioning	Conduct disorder	Substance use	Total
**Connection^1^**	7↑^47 50 51 53^* 55 57 59	6↑^75^† 76 77 78 80 82 1↓‡^81^	4↑^69–71 74^	3↑^63 66 68^			20↑^47^ ^50^ ^51^ ^53^* 55 57 59 63 66 68–71 74–78 80 821↓‡^81^
**Connection^2^**: general communication	1↑^48^						1↑^48^
**Connection^3^** : sexual and reproductive health communication	4↑^55–58^ 1↓^51^ 2↔^52 60^						4↑^55–58^ 1↓^51^ 2↔^52 60^
**Positive behaviour control^1^** (monitoring)	8↑^52 56–62^	1↑^79^	2↑^69 70^	1↑^63^	1↑^49^	2↑^54 90^	15↑^49 52 54 56–63 69 70 79 90^
**Negative behaviour control^2^** (harsh)	1↓^62^	2↓^76 79^	2↓^72 73^	2↓^66 68^	4↓^84–87^ 1(+C^1^)↑^83^ 1(-C^1^)↓^88^	1**↓** 89	12↓^62 66 68 72 73 76 79 84–87 89^ 1(+C^1^)↑^83^ 1(-C^1^)↓^88^
**Role modelling** (of positive or negative behaviour)			1↑^70^(positive modelling for education)			2↓^54 90^(negative modelling of substance use)	1↑^70^(positive modelling) 2↓^54 90^(negative modelling)
**Provision and protection**			1↑^69^	1↑^63^			2↑^63 69^
**Parenting style:** authoritarian	1↑^57^	1↓^81^†		2↓^64 67^			1↑^57^ 3↓^64 67 81^†
**Parenting style:** authoritative	1↑^57^			3↑^64 65 67^			4↑^57 64 65 67^
Total studies (44)	14	8	6	6	7	3	44

+C^1^; with connection (warmth).

-C^1^; without connection (warmth).

positive outcome↑, negative outcome↓, inconsistent↔.

*For girls only.

†Indicates maternal.

‡Indicates paternal warmth.

**Table 4:** Synthesis of results according to parenting exposure: higher quality studies (score >4) (n=18)

Parenting role	Sexual and reproductive health	Mental health	Educational achievement	Adaptive functioning	Conduct disorder	Substance use	Total
**Connection^1^**	3↑^47 50 59^	3↑^75^*^76 77^		1↑^66^			7↑^47 50 59 66 76 77^ ^75^*
**Connection^2^** general communication	1↑^48^						1↑^48^
**Connection^3^** sexual and reproductive health communication	1↔^52^						1↔^52^
**Positive behaviour control^1^** (monitoring)	4↑^52 59 61^ ^62^					1↑^54^	5↑^52 54 59 61 62^
**Negative behaviour control^2^** (harsh).	1↓^62^	1↓^76^	1↓^72^	1↓^66^	2↓^85 87^ 1(+C^1^)↑^83^ 1 (-C^1^)↓^88^	1↓^89^	7↓^62 66 72 76 85 87 89^ 1(+C^1^)↑^83^ 1(-C^1^)↓^88^
**Role modelling** (of positive or negative behaviour)						1↓^54^^(^negative modelling of substance use)	1↓^54^(negative modelling)
**Provision and protection**							
**Parenting style:** authoritarian							
**Parenting style:** authoritative							
Total studies (18)	7	3	1	1	4	2	18

+C^1^; with connection (warmth).

-C^1^; without connection (warmth).

Positive outcome↑, negative outcome↓, inconsistent↔.

*Indicates maternal.

**Table 5:** Synthesis of results according to parenting exposure: poor-medium quality studies (score ≤4) (n=26)

Parenting role	Sexual and reproductive health	Mental health	Educational achievement	Adaptive functioning	Conduct disorder	Substance use	Total
**Connection^1^**	4↑^51 55 57^ ^53^*	1↓^81^† 3↑^78 80 82^	4↑^69–71 74^	2↑^63 68^			1↓^81^† 13↑^51 53^* 55 57 63 68–71 74 78 80 82
**Connection^2^** general communication							
**Connection^3^** sexual and reproductive health communication	4↑^55–58^ 1↓^51^ 1↔^60^						4↑^55–58^ 1↓^51^ 1↔^60^
**Positive behaviour control^1^** (monitoring)	4↑^56–58 60^	1↑^79^	2↑^69 70^	1↑^63^	1↑^49^	1↑^90^	10↑^49 56–58 60 63 69 70 79 90^
**Negative behaviour control^2^** (harsh)		1↓^79^	1↓^73^	1↓^68^	2↓^84 86^		5↓^68 73 79 84 86^
**Role modelling** (of positive or negative behaviour)			1↑^70^(positive modelling for education)			1↓^90^(negative modelling of substance use)	1↑(positive modelling)^70^ 1↓(negative modelling)^90^
**Provision and protection**			1↑^69^	1↑^63^			2↑^63 69^
**Parenting style:** authoritarian	1↑^57^	1↓^81^‡		2↓^64 67^			1↑^57^ 3↓^64 67 81^‡
**Parenting style:** authoritative	1↑^57^			3↑^64 65 67^			4↑^57 64 65 67^
Total studies	7	5	5	5	3	1	26

Positive outcome↑, negative outcome↓, inconsistent↔.

*For girls only.

†Indicates paternal warmth.

‡Indicates maternal.

## Results

### General overview of the studies

There were 44 studies that met our inclusion criteria, the majority of which were cross-sectional (n=35) and there were 9 longitudinal studies. Studies ranged in sample size from 30 to 31 098 and were conducted in 13 SSA countries, with most conducted in Kenya, Ghana and South Africa. Some drew on large-scale national studies including: ‘Transition to Adulthood’ (TTA)^47–49^ and the ‘Cameroon Family and Health Surveys’ (CFHS).^50 51^ Other studies reported findings from more than one SSA country.^52–54^ A small number of studies (n=8) involved younger children, <10 years of age, but the majority were for older children/adolescents. In all cases we refer to ‘parent–child’ relationships. The majority of studies were of poor-medium quality (n=26) and 18 were of higher quality. Most studies were ‘poor-medium’ because of study design (cross-sectional vs longitudinal) and/or because of limitations in parenting (exposure) and child outcome measurements. Most studies reported significant association data from multivariate analysis; however, a small number of studies reported bivariate analysis only (7/44). The main types of confounding factors controlled for across the studies were gender (sex), age and poverty (or socioeconomic status). Approximately half (24/44) of the studies reported local cultural adaptation of parenting measures and included details on translation/back translation and/or piloting to check acceptability and understanding with local samples. The majority of studies reported internal consistency Cronbach alpha values for exposure and outcome measurement tools used. The 44 studies are initially summarised according to the association of parenting with the following child outcomes: (1) SRH —14 studies, (2) adaptive functioning/development—6 studies, (3) educational outcomes/academic achievement—6 studies, (4) mental health/depression—8 studies, (5) ASB/conduct disorders/delinquency—7 studies and (6) substance use (alcohol and tobacco)—3 studies.(online supplementary table 1.1-1.6 in)

### Parenting and SRH

There were 14 studies on parenting and association with child/adolescent risky sexual behaviours. There was inconsistency in the definitions and dimensions of parenting that were measured. Fako^55^ assessed parenting that was measured by a 76-item questionnaire,^55^ whereas Negeri^56^ used six items from Silverberg’s parental monitoring scale and parent–child communication about HIV/AIDS was measured using a five-item scale.^56^ Okigbo *et al* 48 measured general supportive communication, not communication specifically about SRH.^48^ Furthermore, two studies measured parenting dimensions (monitoring/connectedness) *as well as* parenting styles (eg, authoritative/authoritarian).^57 58^ There was also a lack of consistency in scales used to measure SRH outcomes. For example, Negeri^56^ measured outcomes using the WHO SRH questionnaire, while Cherie and Berhanie^57^ used a questionnaire adapted from the ‘Youth Risk Behaviour Survey’ (online supplementary table 1.1).^57^

Results showed that parental connection (warmth) was protective against risky sexual health behaviours in seven studies.^47 50 51 53 55 57 59^ Sidze *et al* 47 showed that parental connection was associated with safer sexual behaviour (condom use) in adolescent males.^47^ There was an association between parent–child ‘cross-gender’ *general* communication and delayed transition to sexual debut in the longitudinal study of Okigbo *et al*.^48^ However, results on the associations between parent–child communication about SRH and outcomes were inconsistent, with four studies indicating protective effects,^55–58^ one showing it increases risky SRH behaviour^51^ and two studies showing inconsistent or limited results.^52 60^ Eight studies showed associations between parental monitoring and protection from risky sexual health outcomes.^52 56–62^ Four higher quality studies showed the protective role of parental monitoring on delayed sexual debut or risky sexual behaviour in adolescents.^52 59 61 62^ Marston *et al* 62 reported an association between experience of ‘severe family dysfunction’ (including physical punishment) and early sexual debut in adolescence.^62^ Finally, Cherie and Berhanie^57^ reported associations between the authoritarian or authoritative parenting styles and students’ safer sexual behaviours, but this was a poor-medium quality study.^57^

### Parenting and child adaptive functioning/ development

There were six studies on associations between parenting and child adaptive functioning and development.^63–68^ All studies were of poor-medium quality except for the higher quality longitudinal study of Tomlinson *et al*.^66^

Various measures were used to assess parenting and outcomes across studies: Torimiro *et al* 63 measured 21 different parenting items^63^; two studies used the Parenting Styles and Dimensions Questionnaire (PSDQ) of Robinson *et al* 64 67; and Kritzas and Grobler^65^ used the Parental Authority Questionnaire.^65^ In addition, three studies measured parenting styles (eg, authoritarian, authoritative).^64 65 67^ In the study by Latouf and Dunn,^64^ children’s outcomes (including social behaviour) were scored independently by their teacher.^64^ In the longitudinal study by Tomlinson *et al,* 66 mother:–child interactions were filmed and infant attachment was assessed using the strange situation technique.^66^

Results showed associations between parent–child connection (warmth) and normal child development in three studies which included healthy attachment and pro-social behaviour.^63 66 68^ Conversely, maternal harsh or intrusive behaviour control was associated with poor adaptive functioning.^66 68^ Parental monitoring (positive behaviour control) as well as ‘provision and protection’ were associated with healthy child development/adaptive functioning, but this was a poor-medium quality study.^63^ Two studies reported associations between the ‘authoritarian’ parenting style and negative outcomes, such as ASB^64^ and negative affect.^67^ In contrast, the ‘authoritative’ parenting style was associated with positive child development including pro-social behaviour,^64^ resilience^65^ and positive life goals and aspirations.^67^ Importantly, the prospective longitudinal study by Tomlinson *et al* 66 showed that positive maternal parenting behaviours are associated with healthy child attachment and child adaptive functioning in SSA (online supplementary table 1.2).^66^

### Parenting and child educational outcomes/academic achievement

There were six studies on parenting and associations with child/adolescent academic achievement or educational outcomes. Some authors measured parenting using questions on parenting practices and expectations, while others devised their own questionnaires. Outcomes measured included adolescent academic goal orientation via the Learning Process Questionnaire^69^ and some studies used children’s school grades, attendance and drop-out rates.^70–73^ The studies were of poor-to-medium quality apart from the higher quality study of Sherr *et al*.^72^

Results showed associations between parental connection and parental monitoring on positive educational outcomes including academic achievement.^69–71 74^ However these studies were poor-medium quality and two reported correlation analysis only.^70 74^ There was an association between parental role modelling of positive behaviour towards school and positive child education outcomes.^70^ There was also an association between ‘provision and protection’ with positive educational outcomes.^69^ Recent studies by Sherr *et al* 72 and Pieterse^73^ showed the detrimental effect of harsh parenting on children’s educational outcomes.^72 73^ Importantly, the higher quality longitudinal study by Sherr *et al* 72 provides evidence of temporal association between harsh parental discipline and the reduced likelihood of the child being enrolled in school at follow-up.^72^ Pieterse^73^ reported the association between harsh parenting (physical and emotional maltreatment) and children’s reduced numeracy test scores and increased probability of school drop-out (online supplementary table 1.3).^73^

### Parenting and adolescent mental health/depression

There were eight studies on the associations between parenting practices and child/adolescent mental health outcomes. The studies were mainly of poor-medium quality, but three were of higher quality.^75–77^ Parenting practices were measured across studies using: the Parenting Style Index (in Maepa *et al*, 2015)^78^; a measure of psychological control (in Bradford *et al* 2004)^79^; the ‘Egna Minnen Betrafande Oppfostran’ (in Khasakhala *et al*)^75 80^; the Parental Bonding Instrument of Parker *et al* (in Mashegoane *et al*)^81^ and the Parental Acceptance-Rejection Questionnaire of Rohner and Khaleque (in Bireda).^82^ In the study by Jewkes *et al,* 77 more severe child maltreatment was measured using the childhood trauma questionnaire (of Bernstein *et al*).^77^ Child and adolescent mental health outcomes were assessed using various tools including the Child Depression Inventory, Centre for Epidemiologic Studies Depression Scale or Beck’s Depression Inventory-II.

Results showed that parental connection (warmth) protected young people from poor mental health outcomes in six studies.^75–78 80 82^ Importantly, these included three higher quality studies.^75–77^ However, in one study paternal ‘care’ was associated with an increase in students’ depressive symptoms.^81^ Khasakhala *et al* 75 reported an association between rejecting maternal behaviour and clinical major depressive disorders in youth.^75^ Okello *et al* 76 reported the significant protective effect of parental connection against depression and anxiety in adolescents from a postwar area of Northern Uganda.^76^ Jewkes *et al* 77 reported baseline data from an RCT which showed that a lack of parental connection (emotional attachment) was associated with depression in males and females.^77^ There was an association between harsh parenting and poor mental health outcomes in two studies.^76 79^ The large cross-cultural comparative study by Bradford *et al* 79 reported associations between harsh parenting (psychological control) and youth depression with marked invariance in results across national samples (10 out of 11 countries).^79^ In the study by Okello *et al,* 76 harsh parenting including physical punishment was associated with poor mental health in adolescents.^76^ Finally, Mashegoane *et al* 81 showed an association between maternal ‘authoritarian’ parenting style and an increase in students’ depressive symptoms; however, this was a poor-medium quality study (online supplementary table 1.4).^81^

### Parenting and ASB/delinquency/conduct disorder

There were seven studies on associations between parenting and child or adolescent ASB, conduct disorders or delinquency. Two studies used the Alabama Parenting Questionnaire to assess aspects of harsh parenting.^83 84^ The studies used various tools to measure child maladaptive behaviours including the Strengths and Difficulties Questionnaire^83 85 86^ and the delinquency sub-scale of the Child Behaviour Checklist-Youth Self Report.^87^ Results from the study of Kabiru *et al* 49 showed an association between the protective effect of parental monitoring and lower delinquency, but this was a poor-medium quality study.^49^ Four studies showed associations between harsh parenting (physical punishment/corporal punishment) and child externalising behaviours including ASB and aggression.^84–87^ The higher quality longitudinal study by Waller *et al* 87 used youth self-reported measures and showed that harsh parenting (emotional and physical harm) predicted ASB over time.^87^ The recent study by Skeen *et al* 85 showed that harsh physical and psychological discipline was associated with more behavioural problems in adolescents.^85^ Two further studies documented the association of harsh behaviour control with negative outcomes^83 88^ and one showed the protective effect of connection.^83^ In the study by Mbagaya *et al,* 88 childhood physical abuse was significantly associated with criminal tendencies in the Kenyan and Zambian samples.^88^ Ward *et al* 84 reported an association between corporal punishment and adolescent externalising and internalising symptoms, but this was a lower quality study.^84^ Finally, Hecker *et al* 86 reported on a study with primary school-aged children and showed that corporal punishment in the home was associated with children’s externalising behaviours, including aggression, conduct disorders and delinquency.(online supplementary table 1.5).^86^

### Parenting and substance (alcohol/tobacco) use

There were three studies identified on associations between parenting and substance use in adolescents.^54 89 90^ The higher quality study by Oladeji *et al* 89 reported on data from the Nigerian study of Mental Health and Wellbeing which investigated childhood adversities and lifetime (DSM-IV) disorders that included substance use disorders.^89^ This study reported an association between parental neglect or abuse and increased risk for substance use disorders (alcohol/drug dependency).^89^ Poms *et al* 54 conducted secondary analysis of data from the Global School Based Health Survey (of 27 countries) that included nine SSA countries.^54^ This study reported the association of parental role modelling of tobacco use and children’s tobacco use. Similarly, Hoque and Ghuman^90^ reported an association between parental role modelling of alcohol use and adolescent alcohol use.^90^ Finally, parental monitoring (including clear house rules about alcohol use) was a protective factor for tobacco or alcohol use among students (online supplementary table 1.6)^54 90^

## Synthesis

### Results synthesis using modified WHO typology

Synthesis of results from the 44 studies was conducted according to a modified version of the WHO parenting dimensions (table 2).^46^ Studies were categorised by parenting practice to enable comparison with findings from HICs. This enabled synthesis and summary of results according to parenting exposure across all outcomes (tables 3–5)

### Connection

Parent–child connection (warmth and support) was protective against poor health and psychosocial outcomes in 20 of the studies (table 3). Parent–child connection was associated with protection from risky sexual behaviours in seven studies^47 50 51 53 55 57 59^ and protection from poor mental health in six studies.^75–78 80 82^ However, in one study ‘paternal’ connection (warmth) was associated with an increase in students’ depressive symptoms.^81^ Parent–child connection was associated with positive educational outcomes in four studies,^69–71 74^ and it was associated with normal child development/healthy attachment in three studies.^63 66 68^

Connection was associated with safer sexual behaviour in three higher quality studies (table 4)^47 50 59^. In the higher quality study by Sidze *et al,* 47 parent–child connection was associated with safer sexual behaviour (including condom use).^47^ Parent–child connection was also associated with safer sexual practices in four lower quality studies.^51 53 55 57^ A lack of parent–child connection was associated with depression and suicidality in the higher quality study of Jewkes *et al*.^77^ Connection was also associated with protection from poor mental health in three poor-medium quality studies (table 5).^78 80 82^

Parent–child connection was associated with positive educational outcomes for children in four studies but these studies were all poor-medium quality.^69–71 74^ Finally, parent–child connection was associated with normal child development and healthy attachment.^63 66 68^ The higher quality study by Tomlinson *et al* 66 showed the important association between positive maternal parenting behaviours and healthy child attachment (table 4)^66^.

### Connection: general communication

The construct of ‘parent–child connection’ has also been defined as communication between parent and child^21^ although most studies in SSA have investigated this in relation to SRH outcomes.^10^ In the present review, one higher quality longitudinal study by Okigbo *et al* 48 found an association between parent and child ‘cross-gender’ general communication and a delay in transition to first sex at wave 2 in the TTA study^48^ (table 3).

### Connection: SRH communication

Parent–child communication, specifically about SRH issues, showed mixed results. Four studies showed that SRH communication was protective against risky SRH outcomes in youth^55–58^ but these were all poor-medium quality studies (table 5). One study reported an association between SRH communication and increased risky sexual behaviours^51^ and two reported mixed or inconsistent findings.^52 60^ The study by Dimbuene and Defo^51^ reported an association between parent–child SRH communication and increased risk of sexual debut, but this was a lower quality study.^51^ The study by Kumi-Kyereme *et al* 60 showed limited effects of SRH communication on adolescents’ sexual activities.^60^ The higher quality study of Biddlecom *et al* 52 reported mixed/inconsistent findings on the association of SRH communication with sexual health behaviours across four different SSA countries^52^ (table 4).

### Positive behaviour control

Positive behaviour control, in the form of parental monitoring, was associated with positive health and psychosocial outcomes in 15 studies across the six different outcomes (table 3). Positive behaviour control was associated with protection from risky SRH outcomes in eight studies^52 56–62^ and protection from poor mental health in one study.^79^ Parental monitoring was associated with positive educational outcomes in two studies^69 70^ and with normal child development in one study.^63^ Furthermore, positive behaviour control was protective against conduct disorders^49^ and substance use in adolescents.^54 90^

Positive behaviour control was protective against risky sexual health outcomes in four higher quality studies^52 59 61 62^ (table 4). Tenkorang and Adjei^61^ reported that adolescents who were closely monitored by parents were less likely to transition to first sex.^61^ Interestingly, Kabiru *et al* 49 reported that higher parental monitoring was associated with lower delinquency in a sample of youth from two slums in Nairobi.^49^ In a higher quality study, Poms *et al* 54 reported that high levels of parental monitoring were associated with less tobacco use by children in all (n=9) SSA countries that were included in their study.^54^ Hoque and Ghuman reported the protective association between parental monitoring of alcohol use (including clear house rules about alcohol use) and adolescents’ alcohol use, though this was a poor-medium quality study^90^ (table 5).

### Negative behaviour control

Negative behaviour control or harsh parenting characterised by physical punishment and/or physical/psychological maltreatment was associated with poor child health and psychosocial outcomes in 12 studies (table 3). Harsh parenting was associated with poor SRH outcomes in one study,^62^ poor mental health outcomes in two studies,^76 79^ poor educational outcomes in two studies^72 73^ and poor adaptive functioning in two studies.^66 68^ Harsh parenting was also associated with increased risk for conduct disorders in four studies^84–87^ and was associated with an increased risk of substance use in one study.^89^ The higher quality study of Marston *et al* 62 reported an association between harsh parenting and early sexual debut^62^ (table 4). Harsh behavioural control (punitive, coercive) increased the risk of poor academic and educational outcomes (in two studies^72 73^) and poor adaptive functioning (in two studies^66 68^). Similar associations between harsh parenting and poor educational outcomes were found in one high quality study^72^ and in one poor-medium quality study.^73^

The longitudinal study by Sherr *et al* 72 showed temporal association between harsh parenting (including physical/psychological punishment) and the reduced likelihood of children being enrolled in school at follow-up^72^ (table 4). Pieterse^73^ reported an association between harsh parenting (physical and emotional child maltreatment) and reduced numeracy test scores as well as increased probability of school drop-out.^73^ Harsh behaviour control (physical/psychological) was associated with depression in two studies.^76 79^ Harsh parenting was also associated with increased risk of ASB/delinquency in four studies: two of which were poor-medium quality^84 86^ and two which were higher quality studies.^85 87^ In the higher quality longitudinal study of Waller *et al,* 87 harsh parenting (physical and emotional punishment) predicted ASB over time.^87^ A further two higher quality studies^83 88^ reported on the interaction between connection and harsh physical discipline (on ASB), with one study showing a moderating effect.^83^ In the study by Mbagaya *et al*, severe physical punishment was associated with criminal tendencies in the Zambian and Kenyan samples.^88^ Finally, negative behaviour control (harsh physical punishment) was associated with increased risk for substance use disorders in one higher quality study^89^ (table 4).

### Role modelling

Parental role modelling of behaviour was associated with positive educational outcomes in one study^70^ and with negative health behaviours in two studies^54 90^ (table 3). In the higher quality study by Poms *et al,* 54 parent tobacco use was associated with child tobacco use.^54^ The study by Bojuwoye and Narain^70^ showed that parental role modelling of positive behaviour towards school was associated with academic achievement; however, this was a poor-medium quality study.^70^ In addition, Hoque and Ghuman^90^ reported an association between parental role modelling of alcohol use and adolescent alcohol use.^90^

### Provision and protection

Parental provision and protection is a relatively new parenting construct defined by the WHO as parents’ provision of resources for children’s schooling/education, as well as building social capital/networks (WHO, 2007).^46^ Two studies reported association between parental provision and protection and positive child outcomes: one reported association with positive academic goals^69^; a second reported association with positive child development.^63^ However, these were both low-medium quality, cross-sectional studies (table 5).

### Parenting styles

#### Authoritarian

The authoritarian parenting style was associated with positive child outcomes in one study^57^ and negative outcomes in three studies^64 67 81^ (table 3). Cherie and Berhanie^57^ reported an association between ‘authoritarian’ parenting and students’ safer sexual behaviour.^57^ Authoritarian parenting (maternal) was associated with an increase in students’ depressive symptoms in the study by Mashegoane *et al*.^81^ Two studies showed associations between ‘authoritarian’ parenting style and poor child adaptive functioning.^64 67^ Latouf and Dunn^64^ reported an association between authoritarian parenting and children’s poor social behaviour, while Roman *et al* 67 reported an association between authoritarian parenting and negative affect.^67^ However, both of these studies were poor-medium quality^64 67^ (table 5).

#### Authoritative

The authoritative parenting style was associated with positive child outcomes in four studies (table 3).^57 64 65 67^ However, these were all of poor-medium quality. Cherie and Berhanie^57^ reported an association between authoritative parenting style and students’ safer sexual behaviours.^57^ Latouf and Dunn^64^ showed an association between authoritative parenting and children’s pro-social behaviour.^64^ Finally, studies by Kritzas and Grobler^65^ and Roman *et al* 67 reported associations between authoritative parenting style and healthy child adaptive functioning including resilience^65^ and positive life goals and aspirations.^67^

## Discussion

This review and synthesis has shown that patterns of parenting and child and adolescent health and psychosocial outcomes in SSA are similar to those that have been well documented in HICs. Importantly, the present review differs from previous work since we investigated *several aspects of* parenting and *a range* of child and adolescent health outcomes in SSA countries. Furthermore, we used a modified version of the WHO (2007) parenting dimensions as an appropriate typology to synthesise the results to facilitate comparison with data from HICs.^46^ As far as we are aware, this is one of the first studies to do so.

Previous reviews have been conducted on aspects of parenting and child health and psychosocial outcomes in LMICs.^10–12^ For example, Meinck *et al* 12 conducted a review of all risk factors (including family conflict) for child abuse victimisation, but most studies included were conducted in South Africa or Egypt.^12^ In addition, Frantz *et al* 11 conducted a review of family factors, such as orphanhood and sibling effects in SSA countries, but they did not investigate parenting types or styles.^11^ Furthermore, Bastien *et al* 10 conducted a review on parent: –child communication and risky sexual activity (but no other outcome) in SSA countries.^10^ In their recent study, Sidze and Defo^50^ included a mini-literature review on parenting (connectedness) and adolescent risky SRH behaviour in the introduction to their article.^50^

The present review included 44 studies that were conducted in 13 SSA countries. The sample of studies was complex and diverse with marked inconsistency in the conceptual framing of ‘parenting’ or parenting dimensions and a range of tools were used to measure parenting exposure(s). Therefore, we used a modified version of the WHO (2007) international typology to synthesise the rich set of results.^46^ Overall, data synthesis showed that similar associations were found between parenting practices and child outcomes in SSA compared with those documented for HICs.^21 91^ This is important since there is ongoing debate in the literature about cultural variation in children’s responses to parenting practices, especially in relation to harsh physical discipline/corporal punishment.^35 38 40^ This review shows associations between ‘harsh’ parenting (in the form of physical punishment) and negative child outcomes in SSA, including externalising behaviours, poor school progress and increased school drop-out rates.^73 85 86^

It is important to highlight some studies in the present review which reflect the structural stressors that characterise SSA countries such as extreme poverty and disadvantage; high risk and prevalence of HIV/AIDS and other disease burdens; and areas of political instability or civil conflict. For example, two studies provided evidence of association between parental connection and protection from risky sexual behaviour in large informal settlements (slums) in Nairobi, Kenya.^47 48^ Other studies involved mothers (living with HIV) and their children and reported maternal parenting behaviours that were associated with healthy attachment and pro-social, child development.^66 68^ A further study demonstrated the protective effect of parent–child connection against adolescent anxiety and depression in a sample of adolescents in a postwar area of Northern Uganda.^76^ Therefore, despite challenging socioeconomic contexts, results concur with findings from HICs on aspects of ‘positive’ parenting which are protective for child health and development.^21 91^

There are some limitations to the present study which must be taken into account when interpreting these findings. First, this was not a systematic review. It did not use an exhaustive set of search terms (eg, each SSA country by name), and we did not contact authors in the field. It is therefore possible that some studies were missed. However, the present review and synthesis was designed to address the research question.^44^ Second, due to resource limitations and the timeframe for the study (up to 2016) it was restricted to peer-reviewed literature excluding the ‘grey’ literature. In addition, most of the studies (n=26) were of poor to medium quality, but when results were compared with results from ‘higher’ quality (n=18) studies, similar associations were found in each. Third, most studies included were cross-sectional which means direction of causation between parenting practices and child outcomes cannot be determined, especially in relation to child misconduct and harsh parenting. Finally, since the majority of studies were based on self-report data, there is the possibility of reporting bias.

These limitations should be balanced by some strengths of the current study. First, it is one of the first reviews to investigate patterns of associations between several parenting styles/behaviours with a range of child health and psychosocial outcomes in a number of SSA countries. Second, it is one of the first studies to compare the patterns of associations between parenting and outcomes with those well documented in HICs. Previous work in the SSA context has addressed other factors such as extended family structure, orphanhood or sibling effects but these were outwith the scope of this review. The present study aimed to investigate parent–child interactions which is of particular importance in the context of SSA countries where little evidence on such parenting practices has been documented. Related to this, and to facilitate a meaningful comparison with parenting evidence in HICs, we used a modified version of the WHO (2007)^46^ international typology as a flexible, culturally appropriate tool to synthesise the African studies. This was done not only to enable comparison with HICs, but also to provide a careful reflection of the parenting exposures present in the SSA studies.

There is currently increased interest in the ‘transferability’ of parenting interventions from HICs to LMICs, as well as the criteria required for the development of effective interventions.^1 3 92–94^ Our results show broadly similar patterns of associations between parenting exposure and child outcomes exist in SSA countries as those previously evidenced in HICs, adding to the evidence base in this area of parenting research in LMICs. The summary of evidence presented in this review also contributes to ‘*defining and understanding the problem and its cause’* which is step 1 in the ‘Six steps in Quality Intervention Development’ model.^93^ This study suggests that parenting interventions (or constituent components) from HICs may have a useful role in SSA countries subject to appropriate adaptation. There is now active debate about degrees of cultural adaptation that may be required for successful transferability of parenting interventions across global settings.^95^ It is interesting to speculate that *the programme theory* underpinning an intervention may be transferable from HICs to LICs while context-specific factors may require adaptation.^94–96^ This aligns with the ‘Parenting for Lifelong Health’ parenting programmes such as ‘Sinovuyo Caring Families for Teens’ which has undergone rigorous, iterative development and has recently been evaluated.^97^

As a result of the present review and synthesis of evidence, we make the following recommendations:

1. There is an ongoing need for better designed, higher quality studies on parenting and child outcomes in SSA countries.
2. There is a need for greater consistency in the conceptual and theoretical framing of ‘parenting’ (parenting styles, practices, dimensions) in order to facilitate cross-study comparisons.
3. There is a need for greater consistency in the design and application of tools that are used to measure parenting practices in SSA. Crucially, these tools should be validated with local populations in SSA countries.

### Conclusions

This is one of the first reviews of associations between parenting practices and child outcomes in SSA and suggests that associations are broadly similar to those in HICs. However, these findings are exploratory and should be interpreted with caution until evidence from higher quality studies is available. Parenting and child health is currently a global policy priority as highlighted by the WHO (2010)^5^ and the Sustainable Development Goals which include early childhood development.^98^ An improved knowledge and evidence base would help stakeholders involved in intervention development in LICs (including transfer and adaptation from HICs) invest limited resources in those programmes most likely to be effective. This is of crucial importance in low-resource areas of the world where they are needed the most, in particular, SSA.

**Key questions**

**What is already known?**

- Evidence from high-income countries (HICs) shows that ‘positive’ parenting practices, including connection, monitoring and modelling appropriate behaviour, enhance children’s development and health.
- The transfer of parenting interventions from HICs to sub-Saharan Africa (SSA) assumes that associations between parenting practices and child outcomes are similar across contrasting regions, but this is currently unknown.

**What are the new findings?**

- There are clear associations between both parental connection and positive behavioural control (monitoring) and positive child outcomes in SSA.
- There are clear associations between negative behavioural control or ‘harsh’ parenting and negative child outcomes in SSA.
- The associations between parenting practices and child health outcomes in SSA are broadly similar to those documented in HICs.
- There are very few high-quality longitudinal studies providing evidence of associations between parenting practices and child outcomes in SSA.

**What do the new findings imply?**

- Parenting interventions developed in HICs may have a useful role in SSA, subject to adaptations for local settings and context.
- There is a need for higher quality studies to build the evidence base on parenting and child outcomes in SSA.
