# Supplementary material for: Are parenting practices associated with the same child outcomes in sub-Saharan African countries as in high-income countries? A review and synthesis
Source: BMJ Glob Health. 2018 Dec 27;3(6):e000912. doi: 10.1136/bmjgh-2018-000912 (PMC6326425; doi:10.1136/bmjgh-2018-000912)
Supplement: Supplementary data [file bmjgh-2018-000912supp001.pdf]

Supplementary Table 1.1: Parenting and SRH in Sub-Saharan Africa

Study (Authors, Year); Location; Design	Sampling Procedure (& Sample)	Parenting Variables	WHO Parenting Dimensions	Child/ Adolescent Health Outcomes	Main findings (significant at P<0.05)	QA
47. Sidze EM et al. (2015) Kenya. Longitudinal	689 sexually experienced 12-22 yr olds (61%M) from Nairobi informal settlements. Random sub-sample Wave 2 Transition to Adulthood study.	Young person's assessment (low, medium, high) of: (i) quality of parent-child connectedness, (ii) parental monitoring.	Connection<br>Positive behaviour control	Sexual activity in last 12 months; multiple sexual partners; condom use at last sex.	Adolescent males with medium or high parental connection more likely to have used a condom at last sex.	9
48. Okigbo CC et al. (2015) Kenya. Longitudinal	Random sample 2 informal settlements, Nairobi. 1,927 unmarried 12-19 yr olds (52% M) at Wave 1 Transition to Adulthood study.	Parental monitoring – of whereabouts. General communication. Discipline: did parents/ guardian 1) verbally reprimand or 2) physically hit them?	Connection: General communication<br>Positive behaviour control<br>Negative behaviour control	Transition to first sex between Wave 1 and 2.	'Cross- gender' general communication with parents associated with delay in transition to sex by Wave 2.	10
50. Sidze EM & Defo BK (2013) Cameroon. Longitudinal	447 sexually active, unmarried 15-24 yr olds (227 F). Representative, random sample from Cameroon Family and Health Survey, Bandjoun.	Parent-child relationships measured as parental monitoring, parent-child communication about sexual matters.	Connection*(*quality of parent-child relationship*)<br>Connection: SRH communication<br>Positive behaviour control	Number of sexual partners in last 12 months. Frequency of contraceptive use in last 12 months. Tendency of having occasional, concurrent partners.	Higher quality of parent child relationships, lower odds of: (i) young males having multiple sexual partners (ii) young females having occasional, concurrent sexual partners.	5
51. Dimbuene ZT & Defo BK (2012). West Cameroon. Cross-sectional	1, 182 12-24 yr olds from Bandjoun (52% F). Random representative sub-sample of CFHS.	Youth report on quality of parent-child relationship, parent-child communication about SRH. Parental monitoring of behaviour.	Connection * (*quality of parent-child relationship)*<br>Connection: SRH communication<br>Positive behaviour control	Sexual debut or waiting time to premarital sex.	Quality of parent-child relationships & higher parental monitoring decreased risk of premarital intercourse. Parent-child communication associated with higher risk of sexual debut.	3
52. Biddlecom A et al. (2009). Burkina Faso, Ghana, Malawi, Uganda. Cross-sectional	Two stage cluster design: 9,762 unmarried 15-19 yr olds (4,420 F). (Burkina Faso, 2,948; Ghana, 2,426; Malawi, 2,025, Uganda, 2,363).	Adolescents' perceptions of: Parental monitoring. Two measures of parental communication about SRH 1) who adolescent talked to about sex 2) who they heard from about contraception.	Connection: SRH communication<br>Positive behaviour control	Sexual activity in last year. Contraceptive use at last sex.	Adolescent M (4/4) and F (3/4 countries) with low parental monitoring more likely to be sexually active. Parental monitoring not associated with contraceptive use at last sex. Parent-child SRH communication associated with contraceptive use [Ghanian F /Ugandan M & F]	5
53. Peltzer K (2010). Botswana, Kenya, Namibia, Senegal, Swaziland, Uganda, Zambia, Zimbabwe. Cross-sectional	10,070 school children (13-15 yr olds) from 8 countries, nationally representative samples. Two stage cluster sample design (approx. 50% F in 6/8 countries).	Parental bonding, parental connection, parental supervision.	Connection<br>Positive behaviour control	Risky sexual behaviour. Early sexual debut <15 years old.	Early sexual debut was associated with poor parental connectedness (only in girls).	4
55. Fako TT (2006) Botswana. Cross-sectional	1,294 students (55%F; 57% urban) from secondary schools/ tertiary institutions (12-22+ yrs). Representative sample.	Students' perception of family coherence: psychological bonding with parents, extent to which sex discussed in family.	Connection<br>Connection: SRH communication	Willingness to test for HIV/ AIDS.	Attachment to parents (& grandparents); family emotional support and discussing sex within family associated with willingness to test for HIV/AIDS.	3
56. Negeri EL (2014) Ethiopia. Cross-sectional	1,183 15-24 yr olds (783 M) in west. Random sample of in-school (600) and out-of-school (583) youth.	Youth reported 1) Parental monitoring (Silverberg's parental monitoring scale) 6 items, 2) Parent-adolescent communication scale.	Connection: SRH communication<br>Positive behaviour control	Sexual risk behaviour; substance use; consistent condom use.	Youth with parental monitoring less likely to have had premarital sex. Odds of having had sex 2 x higher among youths who didn't discuss sexual matters with parents.	3
57. Cherie A & Berhanie Y (2015) Ethiopia. Cross-sectional	3,543 randomly selected Addis Ababa high school students (1, 789 F) age 15-24 yrs.	Parent-adolescent communication, parental monitoring, parental connectedness, parenting styles.	Connection<br>Connection: SRH communication<br>Positive behaviour control<br>Parenting styles	Risky sexual behaviour (Youth Risk Behaviour Survey). (youth reported)	Students' safer sexual behaviour associated with parental connectedness, parental monitoring, authoritarian parents, authoritative parents & parents who communicated with them about SRH.	3
58. Dessie Y et al. (2014). Ethiopia. Cross-sectional	633 13-18 yr olds with sexual initiations from Harar (327F). 81% 'in-school'. Random sample, community based study.	Parental monitoring, SRH communication. Parenting styles measured via 2 questions on adolescents' perceptions of parents' supportiveness (warmth) and strictness (control).	Connection: SRH communication<br>Positive behaviour control<br>Parenting styles	Risky sexual practices 1) Not using condom/contraceptive at last sex, 2) Multiple sexual partners in last 12 months.	High parental monitoring decreased risky sexual practices. Satisfactory level of adolescent-parent SRH communication decreased ┼(marginal) risky sexual practices. No association between parenting styles and risky sexual practices.	3
59. Dimbuene ZT & Defo BK (2011). West Cameroon. Cross-sectional	1,025 sexually experienced 12-24 yr olds from Bandjoun (55% F). Representative sample.	Quality of parent-child relationships, parent-child communication about sex, parental control.	Connection * (*quality of parent-child relationship)*<br>Connection: SRH communication<br>Positive behaviour control	Risky sexual behaviour.	Quality of parent/guardian - youth relationships and parental control decrease the odds of risky sexual behaviour.	5
60. Kumi-Kyereme A et al. (2007) Ghana.	4,430 unmarried 12-19 yr olds (2, 229 M).	Communication about SRH, types of people who talked to adolescents about	Connection: SRH communication<br>Positive behaviour control	Sexual activity in last 12 months.	Negative association between parental monitoring and adolescents' sexual activity (M	3

Supplementary Table 1.1: Parenting and SRH in Sub-Saharan Africa						
Study (Authors, Year); Location; Design	Sampling Procedure (& Sample)	Parenting Variables	WHO Parenting Dimensions	Child/ Adolescent Health Outcomes	Main findings (significant at P<0.05)	QA
Cross-sectional		SRH, parental monitoring.			& F). Limited effects of communication.	
61. Tenkorang EY & Adjei JK (2015) Ghana. Cross-sectional	2,387 12-19 yr olds (51%M). Stratified sample (urban & rural).	Parental monitoring, communication (about sex-related issues).	Connection: SRH communication<br>Positive behaviour control	Transition to first sex.	Adolescents closely monitored by parents less likely to transition to first sex.	5
62. Marston M et al. (2013) Kenya. Longitudinal	Random sample, informal settlements, Nairobi. 1,754 sexually inexperienced 12-16 yr olds (at wave 1). 92 experienced early sexual transition (53 M).	Supervision (parental monitoring). Parental connection (support). Family dysfunction (parent jobless/ adult ever hit you).	Connection<br>Positive behaviour control<br>Negative behaviour control	Early sexual debut; self-esteem.	Lack of parental supervision was predictor of sexual debut (M only). For both M & F, transition to first sex associated with "having experienced severe family dysfunction."	8

Supplementary Table 1.2: Parenting and Child Adaptive Functioning (Development) in Sub-Saharan Africa						
Study (Authors, Year); Location	Sampling Procedure (& Sample)	Parenting Variables	WHO Parenting Dimensions	Child/ Adolescent Health Outcomes	Main findings (significant at P<0.05)	QA
63. Torimiro DO et al. (2004). Nigeria. Cross-sectional	186 parents from rural South West/ Oyo state (85% M) and their children (11-18 yr olds, 63% M). Multi-stage/ random sampling.	Parental reports about parent and child. Measured 21 aspects of parenting roles including discipline, support, guidance.	Connection<br>Positive behaviour control<br>Provision and protection	Child development. Child cognitive, social, physical, emotional and moral development.	Positive relationship between level of parenting role performance and child development.	0
64. Latouf N & Dunn M (2010). South Africa. Cross-sectional	30 parents (15 M) of 25 5 yr old children (16 girls) and their female teacher from Northern Province school. Convenience sample.	Parents completed the Parental Styles Dimensions Questionnaire (PSDQ: Robinson *et al* 2001) for self and spouse.	Parenting styles: Authoritarian, Authoritative, Permissive.	Teacher observation of child behaviour. Aggression; disrespect; hyperactivity; detachment. Pro-social behaviour & anti-social behaviour.	Parent-rated (mothers) authoritative parenting style associated with teacher-rated child pro-social behaviour. Parent-rated (mothers) authoritarian parenting style associated with teacher-rated child anti-social behaviour.	4
65. Kritzas N & Grobler, AA (2005). South Africa. Cross-sectional	360 English speaking Grade 12 learners (215 F), mean age 17.6 yrs, from Bloemfontein.	Adolescents completed Parental Authority Questionnaire about mothers and fathers styles: 1) permissiveness, 2) authoritarianism, 3) authoritativeness.	Parenting styles: Authoritarian, Authoritative, Permissive.	Orientation to Life Questionnaire (OLQ) and the Cope Scale (adolescents' coping strategies, sense of coherence and resilience).	Authoritative parenting positively associated with adolescent's resilience. Paternal authoritarian style associated with emotion focused coping strategies (but only in white adolescents).	3
66. Tomlinson M et al. (2005). South Africa. Longitudinal	147 mother-infant dyads from Khayelitsha and 'Town II', Cape Town. Mothers aged < 20-39yrs: infants (53% M). 98 at 18 month follow-up. Representative sample.	Mother-child interactions: responsiveness, acceptance & warmth; coercive, intrusive behaviours; remote, disengaged behaviours.	Connection (lack of)<br>Negative behaviour control	Infant attachment	Indices of poor parenting at 2 and 18 months associated with insecure infant attachment. 2 month predictors of insecure infant attachment were maternal intrusiveness and remoteness.	9
67. Roman NV et al. (2015). South Africa. Cross-sectional	853 Grade 11 public school learners from 8 school districts in Western Cape (486 F), mean age, 17 yrs.	Learners completed the Parenting Style and Dimensions Questionnaire (PSDQ).	Parenting styles: Authoritarian, Authoritative, Permissive.	Adoption of life goals/ aspirations: Psychological Needs Scale (PNS); Aspiration Index (AI); Positive/ negative affect scale (psychological wellbeing).	Authoritative parenting predicted intrinsic life goals/ aspirations. Authoritarian & permissive parenting predicted extrinsic life goals. Maternal & paternal authoritarian and maternal permissive parenting predicted negative affect.	3
68. Allen AB et al. (2014). South Africa Cross-sectional	361 women (22-52 yrs old) from Tshwane (living with HIV) with 6-10 yr old child. From primary health and immunology clinics. Sampling procedure not reported.	Mother reported 1) Parenting Stress Index (PSI) – distress/ parent-child dysfunction, 2) Coping with child's negative emotion scale – positive (e.g. encouragement) vs negative (e.g. punitive) dimensions.	Connection<br>'Parenting style' *but not* Baumrind's – 'positive' (encouragement) vs 'negative' (punitive)<br>Negative behaviour control	Mother reported on child's adaptive functioning: communication, daily living skills, socialisation. Parental report on child's internalising/ externalising behaviour via Child Behaviour Check List (CBCL).	Negative maternal parenting style associated with higher child internalising + externalising behaviour. Positive parenting style associated with lower externalising behaviour, higher socialisation & daily living skills and did not predict internalising behaviours.	4

Supplementary Table 1.3: Parenting and Child Education/ Academic Achievement in Sub-Saharan Africa						
Study (Authors, Year); Location	Sampling Procedure (& Sample)	Parenting Variables	WHO Parenting Dimensions	Child/ Adolescent Health Outcomes	Main findings (significant at P<0.05)	QA
69. Marjoribanks K & Mboya M (2000). South Africa. Cross-sectional	845 secondary school students (435F) from 5 rural schools (497) and 3 urban schools (348) in rural Eastern Cape & urban Metropolitan Cape. Mean age	Parents' support for learning (19 items) related to 1) parenting style 2) parents' educational expectations 3) parenting practices.	Connection<br>Positive behaviour control<br>Provision and protection	Self-Description Inventory (SDI) for African students (measures adolescent self-concept). Learning Process Questionnaire	Parents' support for learning associated with adolescents' academic goal orientations.	4

Supplementary Table 1.3: Parenting and Child Education/ Academic Achievement in Sub-Saharan Africa						
Study (Authors, Year); Location	Sampling Procedure (& Sample)	Parenting Variables	WHO Parenting Dimensions	Child/ Adolescent Health Outcomes	Main findings (significant at P<0.05)	QA
	18.3 yrs. Sampling procedure not reported.			(measures academic goal orientation).		
70. Bojuwoye O & Narain M (2008). South Africa. Cross-sectional	115 parents (90 F) and their 115 15-18 yr old children (77 F) from 4 Durban schools. Sampling procedure not reported.	Parent reported: parental aspirations for child's education; parent-child communication about school; parental involvement at home; school-home communication; parents' participation in school-related activities.	Connection<br>Positive behaviour control<br>Role modelling	Formal school results: academic achievement.	Parent's involvement in their children's education positively associated with children's academic achievement.	2
71. Cherian VI (1993). South Africa Cross-sectional	1,021 urban & rural black Xhosa speaking 13-17 yr olds (652 F). Standard 7, from Transkei. Stratified random sample.	Parents' interest in child's education: parents' 1) discussion of education with child 2) encouragement 3) help with studies.	Connection	Standard achievement tests/ exam scores at end of standard 7.	Positive association between parental interest and children's academic achievement (monogamous or polygynous families).	4
72. Sherr L et al (2016). South Africa, Malawi. Longitudinal	989 4-13 yr olds (503 F) and their primary caregiver attending community based organisations. Baseline and 15 month follow up. Stratified random sample.	Carer reported: harsh discipline (punishment at home) with Parent Child Conflict Tactics Scale & International Society for Prevention of Child Abuse and Neglect (ISPCAN) screening tools (psychological/ physical violence).	Negative behaviour control (harsh)	Carer reported educational outcomes. Including school: enrolment, attendance, progress.	*Baseline*: physical violence/ harsh discipline associated with lower odds of being in the correct class. *Follow up*: children who experienced psychological violence as discipline >10 x less likely to be enrolled in school. For HIV +ve children, physical violence as discipline associated with poor school performance.	10
73. Pieterse D (2015). South Africa. Longitudinal	4,747 14-22 yr olds (52% F) from Cape Town. Random sample (Wave 1).	Young people's report: experience of physical and emotional violence by parents in the home (aggregate score).	Negative behaviour control (harsh)	Education outcomes, including: numeracy scores and school drop-out.	Physical and emotional maltreatment associated with reduction in numeracy. Significant (& large) *effect* of childhood maltreatment on probability of school drop-out.	4
74. Mboya M (1996). South Africa Cross-sectional	1,192 14-20 yr olds (631 F) from 3 secondary schools in Cape Town Metropolitan area (extremely impoverished).	Students' perceptions – Perceived Parental Behaviour Inventory (PPBI): parents' support & expectations about school, parental participation in school work.	Connection	Self-Description Inventory (SDI) for African students (measures adaptive behaviours, general school/ global self-concept).	Parental support, interest, encouragement & expectations about school were predictors of African adolescents' global self-concept & general school self-concept.	3

Supplementary Table 1.4: Parenting and Mental Health (Depression) in Sub-Saharan Africa						
Study (Authors, Year); Location	Sampling Procedure (& Sample)	Parenting Variables	WHO Parenting Dimensions	Child/ Adolescent Health Outcomes	Main findings (significant at P<0.05)	QA
75. Khasakhala LI et al. (2013). Kenya. Cross-sectional	245 purposefully selected 13-22 yr olds (100 F) attending Nairobi psychiatric outpatient clinic and parents (428: 226 mothers; 202 fathers).	Youth report 'EMBU' (own memories of childhood upbringing): parental-emotional attachment, rejecting behaviour, under-protective behaviour, authoritative behaviour.	Connection (*lack of*)	Youth presenting with major depressive disorders (MDD). Psychiatric disorders.	Maternal rejecting behaviour associated with major depressive disorder (MDD) in youth.	5
76. Okello J et al. (2014) Uganda. Cross-sectional	551 13-21 yr olds from 7 government and private secondary schools Gulu District, Northern Uganda. Convenience sample.	Adverse Childhood Exposure (ACE) questionnaire (incl. physical abuse). Attachment to/alienation from parents measured with inventory of parent/peer attachment.	Connection<br>Negative behaviour control	'HSCL-37A' used for (self-reported) measures of depression and anxiety.	Paternal & maternal attachment associated with less depression and anxiety in adolescents. Alienation by mothers independently associated with increased depression & anxiety in adolescents.	5
77. Jewkes RK et al. (2010). South Africa. Longitudinal	2,782 16-23 yr olds (1,415 F) from rural Eastern Cape schools. Baseline stratified 2 stage sample, 2 yr follow up.	Young person self-report – Childhood Trauma Questionnaire- emotional neglect/abuse, physical neglect.	Connection (*lack of*)<br>Negative behaviour control (severe)	Depression/ suicidal thoughts (CES-D). Alcohol use – 'AUDIT'.<br>*Follow up* HSV2 /HIV status.	Emotional neglect in women associated with depression, suicidality, alcohol abuse and incident HSV-2 infection. Emotional neglect in men associated with depression and drug use.	11
78. Maepa MP et al. (2015). South Africa, Zimbabwe. Cross-sectional	600 8-18 yr olds: 300 street children (snowball sample) (58% F), 300 non-street children (random sample) (57% F).	Parenting Style Index (PSI) (children's self-report).	Connection (*lack of*)<br>Positive behaviour control (*lack of*)	Child abuse and trauma scale. Used to assess trauma (as outcome).	Parental warmth negatively associated with childhood trauma (homelessness), general abuse. No association between parental supervision & childhood trauma.	1
79. Bradford K et al. (2004). South Africa (3 Ethnic groups); Bangladesh; China;	9,050 school going 14-17 yr olds from 11 nations. Includes 1,743 South Africans: 635 Black; 520 Colored; 579	Parental monitoring of adolescent behaviours. Parental support - subscale of Child Report of Parent Behaviour	Connection<br>Positive behaviour control<br>Negative behaviour control	Internalising/ externalising behaviours: social initiative (pro-social behaviour). Child	Parental support associated with higher youth social initiative (11/11 countries). Psychological control associated with higher	4

**Supplementary Table 1.4: Parenting and Mental Health (Depression) in Sub-Saharan Africa**						
Study (Authors, Year); Location	Sampling Procedure (& Sample)	Parenting Variables	WHO Parenting Dimensions	Child/ Adolescent Health Outcomes	Main findings (significant at P<0.05)	QA
India; Bosnia; Germany; Palestine; USA; Colombia. Cross-sectional	White.	Inventory (CRPBI). Parental psychological control – youth self-reported.		Depression Inventory (CDI). Child Behaviour Checklist (anti-social behaviour).	youth depression (10/11 countries). Behavioural control associated with lower youth anti-social behaviour.	
80. Khasakhala LI et al. (2012). Kenya. Cross-sectional	1,276 13-22 yr olds (747 M) from stratified sample of 17 Nairobi public secondary schools. Random sample.	Adolescent completed 'Egna Minnen Betrafande Uppfostran' (EMBU) (own memories of childhood upbringing).	Connection (*lack of*)	Child Depression Inventory (CDI) for depressive disorders and suicidal behaviours.	No emotional attachment (both parents); maternal rejecting & paternal under-protective behaviour associated with adolescent depressive symptoms & suicidal behaviour.	1
81. Mashegoane S et al. (2007). South Africa. Cross-sectional	522 university students (268 M). Average age 20.3 yrs. Convenience sample.	Students' self-report – Parental Bonding Instrument (PBI)- maternal and paternal behaviours, separately.	Connection<br>Negative behaviour control<br>Parenting styles: Authoritarian, Authoritative	Beck's Depression Inventory (BDI-II)-measures students' depressive symptoms.	Maternal 'authoritarian' & paternal 'care' associated with increase in students' depressive symptoms. Paternal 'protectiveness' associated with decrease in depressive symptoms.	4
82. Bireda AD (2015). Ethiopia**.** Cross-sectional	809 adolescents (427 M), mean age 16.8 yrs. Randomly selected from 4 Addis Ababa private and government high schools.	Adolescent report of parental 'warmth/affection' sub-scale of Child Parental Acceptance Rejection Control Questionnaire (PARQ).	Connection	Rosenberg self-esteem scale; school adjustment; 'CES-D' for depressive symptoms (adolescent completed).	Higher parental 'warmth' associated with: fewer school adjustment problems, decreased adolescent depression & increased self-esteem.	1

**Supplementary Table 1.5: Parenting and Conduct Disorder (ASB) in Sub-Saharan Africa**						
Study (Authors, Year); Location	Sampling Procedure (& Sample)	Parenting Variables	WHO Parenting Dimensions	Child/ Adolescent Health Outcomes	Main findings (significant at P<0.05)	QA
49. Kabiru CW et al. (2014). Kenya. Cross-sectional	3,064 12-22 yr olds from 2 slums, Nairobi. 1,566 M. Random sampling baseline survey of TTA study.	Composite index of severe 'Adverse Life Events'. Parental monitoring.	Positive behaviour control<br>Negative behaviour control<br>*(severe experiences)*	Delinquent behaviour (adolescence).	'Adversity' associated with delinquency. Higher parental monitoring associated with lower delinquency. Interaction between parental monitoring & number of adverse life events	4
83. Casale M et al. (2015). South Africa. Cross-sectional	2,477 10-17 yr olds (54% F) and adult caregiver (89%) dyads, from 1 urban/ 1 rural community, Kwazulu Natal. Stratified systematic random sampling.	Caregiver reports: Alabama Parenting Questionnaire (APQ- short form) sub-scales on: positive parenting, consistency of discipline, caregiver supervision.	Connection<br>Negative behaviour control	Care-giver reported Strengths and Difficulties Questionnaire (SDQ) measures: peer problems, hyperactivity, conduct problems, emotional symptoms, pro-social behaviour.	Carer social support and better parenting were predictors of fewer children/ adolescent: peer problems; hyperactivity, conduct problems & emotional symptoms.	5
84. Ward CL et al. (2015). South Africa. Cross-sectional	220 caregivers reporting on their 213 6-18 yr old children (107 M). Rural community study.	Alabama Parenting Questionnaire (APQ). Parenting Stress Index (PSI) for parent-child dysfunctional interactions.	Negative behaviour control (severe)	Parents completed Child Behaviour Checklist for children's externalising (aggressive) and internalising (anxiety and depression) behaviours.	Corporal punishment was associated with children's internalising and externalising symptoms.	4
85. Skeen S et al. (2016) South Africa, Malawi. Cross-sectional	989 4-13 yr olds affected by HIV (51% F) with their carers. 834 from South Africa, 155 from Malawi. Stratified sampling procedure.	Carer reported harsh discipline practices – measures adapted from Parent-Child Conflict Tactics Scale and from ISPCAN screening tools.	Negative behaviour control (severe)	Child Depression Inventory (CDI); Trauma symptom checklist; Rosenberg self-esteem scale; Strengths & Difficulties Questionnaire (SDQ) (internalising +externalising problems). Adolescents reported on their risk behaviours.	Harsh physical & psychological discipline associated with lower self-esteem and more behavioural & emotional problems in adolescents.	5
86. Hecker T et al. (2014) Tanzania. Cross-sectional	409 6-15 yr olds (mean age, 10.5 yrs), grade 2-7 (52% M). From (private) primary school, southern Tanzania.	Children's report on corporal punishment by parents/carers-assessed by 4 questions.	Negative behaviour control<br>*(severe/ harsh)*	Reactive Proactive Questionnaire (RPQ)-current/lifetime aggressive behaviour. Strengths & Difficulties Questionnaire (SDQ) internalising + externalising problems (self-report).	Corporal punishment positively associated with children's current/ lifetime aggressive behaviour, conduct problems and hyperactivity. Prosocial behaviour negatively associated with corporal punishment.	4
87. Waller R et al. (2014). South Africa. Longitudinal	3, 515 10-17 yr olds (1,992 F) from 2 provinces (rural & urban) western Cape & Mpumlanga. 3,401 (97%) retained at 1 year follow up (57% F). Stratified	Harsh home/community environments. Youth self-report of physical and emotional abuse (UNICEF scales).	Negative behaviour control (severe)	Youth anti-social behaviour measured via delinquency sub-scale of 'CBCL-YSR'. Substance use (self-report).	Child emotional and physical harm predicted anti-social behaviour (ASB) over time.	10

Supplementary Table 1.5: Parenting and Conduct Disorder (ASB) in Sub-Saharan Africa						
Study (Authors, Year); Location	Sampling Procedure (& Sample)	Parenting Variables	WHO Parenting Dimensions	Child/ Adolescent Health Outcomes	Main findings (significant at P<0.05)	QA
	random sampling.					
88. Mbagaya C et al. (2013). Kenya, Zambia, Netherlands. Cross-sectional	862 university students (18-40 yr olds). 375 Kenyan, 182 Zambian, 305 Netherlands.	Self-reported childhood physical abuse and neglect. Child physical abuse scale of the Personal and Relationships profile.	Connection (*severe lack of*) Negative behaviour control (severe/punitive)	Depressive symptoms, criminal tendencies, psychopathology, borderline personality disorders.	Childhood 'neglect' associated with depressive symptoms (3 countries). Physical 'abuse' associated with criminal tendencies in Kenyan & Zambian samples.	5

Supplementary Table 1.6: Parenting and Substance Use in Sub-Saharan Africa						
Study (Authors, Year); Location	Sampling Procedure (& Sample)	Parenting Variables	WHO Parenting Dimensions	Child/ Adolescent Health Outcomes	Main findings (significant at P<0.05)	QA
54. Poms LW et al. (2012).Incl. Botswana, Kenya, Mauritius, Namibia, Senegal, Seychelles, Tanzania, Uganda, Zimbabwe. Cross-sectional	106,041 middle school students (13-15 yr olds) from 27 countries, including 31,098 from 9 SSA countries. 2 stage cluster design, representative sampling.	Students' perceptions of: parents' tobacco use; parents 'understanding' and 'awareness' of children's activities.	Connection Positive behaviour control Role modelling	Tobacco use (adolescent report).	Parental tobacco use associated with child tobacco use. Higher levels of parental 'understanding' (7/9 SSA countries) and monitoring (9/9 SSA countries) were associated with less tobacco use by children.	5
89. Oladeji BD et al. (2010). Nigeria. Cross-sectional	2,143 adults, >50% aged <35 yrs (51% F). Range 18≥75 yrs. Multi-stage random sampling.	'Adverse Childhood Experiences'- neglect or abuse in family (being hit or unsupervised in childhood).	Connection (*lack of*) Positive behaviour control *(lack of)* Negative behaviour control *(severe)*	Lifetime mental health disorders (incl. substance use disorders).	Childhood neglect or abuse associated with increased risk of substance use disorders (alcohol/drug dependency).	5
90. Hoque M & Ghuman S (2012). South Africa. Cross-sectional	704 16-18 yr old students (421 F) from 5 Kwazulu Natal high schools. Convenience sample.	Students' perceptions of: house rules about alcohol consumption; parental awareness and communication (about alcohol); parental alcohol behaviour.	Positive behaviour control Role modelling	Alcohol use (adolescent report).	Association between parents' & adolescent alcohol use. Adolescents more likely to use alcohol if ever seen parents drunk. Clear house rules protective factor for alcohol use among students.	3

Supplementary Tables 1.1-1.6: Data Extraction Tables
